# The Effectiveness of Auricular Acupuncture on the Levels of Cortisol in a Depressed Patient

**Published:** 2019-09

**Authors:** Bijan PIRNIA, Negin MOHAMMADZADEH BAZARGAN, Mostafa HAMDIEH, Kambiz PIRNIA, Parastoo MALEKANMEHR, Faezeh MALEKI, Alireza ZAHIRODDIN

**Affiliations:** 1. Department of Psychology, Faculty of Humanities, University of Science and Culture, Tehran, Iran; 2. Department of Psychiatry, Behavior Research Center, Shahid Beheshti University of Medical Sciences, Tehran, Iran; 3. Department of Psychology, Science and Research Branch, Islamic Azad University, Tehran, Iran; 4. Department of Psychosomatic, Taleghani Hospital, Faculty of Medicine, Shahid Beheshti University of Medical Sciences, Tehran, Iran; 5. Bijan Center for Substance Abuse Treatment, Tehran, Iran; 6. Department of Psychology, Hamedan Branch, Islamic Azad University, Hamedan, Iran; 7. Department of Psychology, Tabriz Branch, Islamic Azad University, Tabriz, Iran

## Dear Editor-in-Chief

Suicide among patients with major depressive disorder has considerable frequency (1). On the other hand, the suicide rate in patients treated with methadone treatment is several times that of the general population.

One of the theories considered in recent years is the theory of neural plasticity. Irregularities of the hypothalamic-pituitary-adrenal (HPA) axis leads to depression cognitive symptoms and could be involved in the pathophysiology of suicidal behavior (2). High levels of cortisol have been reported in suicide attempters.

**Fig. 1:**
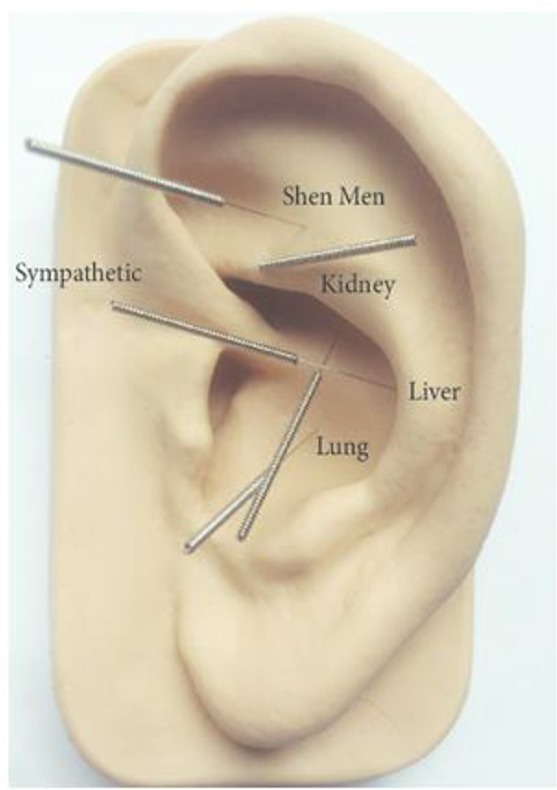
Acupuncture points according to the NADA protocol

Acupuncture is considered as a complementary therapy in medicine that has two thousand years old (3). National Acupuncture Detoxification Association (NADA) is an applied Manuel of Protocol of auricular acupuncture (Fig. 1). Several studies have been conducted on the effectiveness of acupuncture on cortisol level (4, 5). Acupuncture has been associated with the regulation of HPA activity, improving the performance of adrenocorticotropic hormone and controlling of cortisol secretion (4).

The aim of this study was to evaluate the effectiveness of auricular acupuncture on the secretion of saliva cortisol.

This is single-subject research in the form of A-B pattern with multiple baseline design (IBM SPSS Statistics Ver. 20, IBM Corp., Armonk, NY, USA).

A patient was a 32-year-old man with a history of multiple courses of major depression. He had a marriage led to separation at age 21 yr that ruminating thoughts of infidelity is still ongoing. Heroin abuse was started from the age of 23 and after two years, he tried injection consumption pattern. The patient had a history of three times suicide attempts. Two-suicide committing were done with high doses of benzodiazepines and the last one is taken by cutting the veins of the left hand in the camp. Moreover, the patient for depression treatment treated with venlafaxine at a dose of 225 mg daily. Drug treatment was stopped after a period of schema therapy. Now for 3 months, he is under treatment with methadone maintenance in the form of syrup with a daily dose of 8 mg and he does not consume any psychiatric drug.

Acupuncture was performed twice a week for four weeks (twice per week) and was performed in both ears using disposable stainless steel needles (0.25+13 mm) with a depth of 2–3 mm.

Cortisol was evaluated two days a week randomly by Salvi tests and ELISA. The cortisol samples were stored at −20 °C or below until the evaluation. The informed consent was obtained and the whole process was carried out based on the latest version of the Declaration of Helsinki (6).

**Fig. 2:**
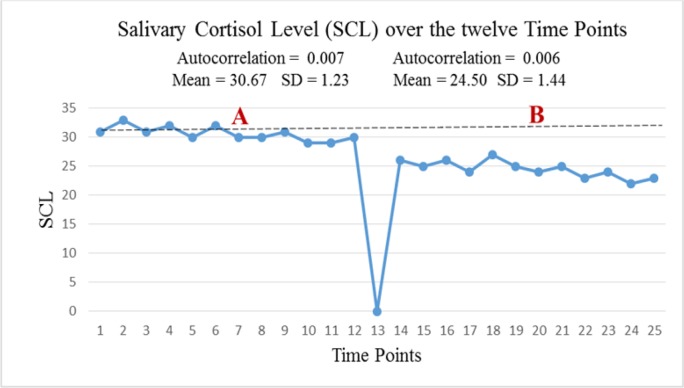
Distribution of salivary cortisol in sixteen assessments stages during Twelve weeks

The secretion rate of cortisol during eight sessions (six weeks) of baseline (left side) and during eight sessions of acupuncture (right side) is presented in Fig. 2.

As it can be seen in Fig. 1, in cortisol level, auto-correlation between the measurement points at baseline was calculated equal to 0.007 and 0.006 in the treatment stage (*P*<0.01) and in suicidal ideation, was calculated equal to 0.005 and 0.008 in the treatment stage (*P*<0.01). The evaluation of urine during twelve weeks indicate patient adherence to abstinence from opioids use in the treatment process (*P*<0.05).

Primary outcomes showed that 4 wk of acupuncture decreased salivary cortisol. Secondary outcomes also showed that acupuncture was associated with a reduction in suicidal ideation. In line with the primary outcomes of the present research, the use of acupuncture through promoting adrenocorticotropic hormone and the secretion of plasma cortisol led to regulation of HPA axis activity in the mice with asthma (4, 5, 7).

The secondary results of this study showed that acupuncture reduced the severity of suicidal ideation. These results are consistent with the findings of another study (5). In line with the findings of the present study, electro-acupuncture by modulating the HPA axis function and enhancing the activity of the hippocampus was associated with reduced symptoms of depression (8). Results of this study showed that auricular acupuncture could be an alternative therapy for medication and psychotherapy in reducing suicidal ideation and improving HPA axis function by modulating secretion of cortisol. Future studies could investigate drug treatments affecting the HPA axis in order to manage suicide.
